# Diagnostic Accuracy of Shear Wave Elastography Versus Ultrasound in Plantar Fasciitis Among Patients with and Without Ankylosing Spondylitis

**DOI:** 10.3390/diagnostics15151967

**Published:** 2025-08-05

**Authors:** Mahyar Daskareh, Mahsa Mehdipour Dalivand, Saeid Esmaeilian, Aseme Pourrajabi, Seyed Ali Moshtaghioon, Elham Rahmanipour, Ahmadreza Jamshidi, Majid Alikhani, Mohammad Ghorbani

**Affiliations:** 1 Department of Radiology, University of California San Diego, San Diego, CA 92093, USA; mahyar.daskareh@hotmail.com; 2 Department of Radiology, Hospital of the University of Pennsylvania, Philadelphia, PA 19104, USA; 3 Guilan Rheumatology Research Center, Department of Rheumatology, Razi Hospital, School of Medicine, Guilan University of Medical Sciences, Rasht 41346-14335, Iran; drmahsamehdipour@gmail.com; 4 Medical Imaging Research Center, Shiraz University of Medical Sciences, Shiraz 71348-14336, Iran; kmssd87@gmail.com; 5 Cardiovascular Diseases Research Center, Department of Cardiology, Heshmat Hospital, School of Medicine, Guilan University of Medical Sciences, Rasht 41448-95655, Iran; aseme.pourrajabi.ap@gmail.com; 6 Department of Orthopedic and Trauma Surgery, Shariati Hospital, Tehran University of Medical Sciences, Tehran 14117-13137, Iran; a_moshtaghioon@yahoo.com; 7 Immunology Research Center, Mashhad University of Medical Sciences, Mashhad 91779-48564, Iran; elhamrahmanipour@gmail.com; 8 Department of Internal Medicine, School of Medicine, Rheumatology Research Center, Shariati Hospital, Tehran University of Medical Sciences, Tehran 14117-13137, Iran; jamshida@sina.tums.ac.ir; 9 Orthopedic Research Center, Department of Orthopedic Surgery, Mashhad University of Medical Sciences, Mashhad 91379-13131, Iran

**Keywords:** shear wave elastography, Belgrade ultrasound enthesitis score, plantar fasciitis, ankylosing spondylitis

## Abstract

**Background:** Plantar fasciitis (PF) is a common enthesopathy in patients with ankylosing spondylitis (AS). Shear wave elastography (SWE) and the Belgrade ultrasound enthesitis score (BUSES) may detect PF, but their comparative diagnostic performance is unclear. **Objective:** To compare SWE with the BUSES for identifying PF in individuals with and without AS. **Methods:** In this cross-sectional study, 96 participants were stratified into AS and non-AS populations, each further divided based on the presence or absence of clinical PF. Demographic data, the American Orthopedic Foot and Ankle Society Score (AOFAS), and the BASDAI score were recorded. All subjects underwent grayscale ultrasonography, the BUSES scoring, and SWE assessment of the plantar fascia. Logistic regression models were constructed for each population, controlling for age, body mass index (BMI), and fascia–skin distance. ROC curve analyses were performed to evaluate diagnostic accuracy. **Results:** In both AS and non-AS groups, SWE and the BUSES were significant predictors of PF (*p* < 0.05). SWE demonstrated slightly higher diagnostic accuracy, with area under the curve (AUC) values of 0.845 (AS) and 0.837 (non-AS), compared to the BUSES with AUCs of 0.785 and 0.831, respectively. SWE also showed stronger adjusted odds ratios in regression models. The interobserver agreement was good to excellent for both modalities. **Conclusions:** Both SWE and the BUSES are effective for PF detection, with SWE offering marginally superior diagnostic performance, particularly in AS patients. SWE may enhance the early identification of biomechanical changes in the plantar fascia.

## 1. Introduction

Plantar fasciitis (PF) is a prevalent condition characterized by heel pain and functional impairment, affecting individuals across diverse age groups and activity levels [1]. It involves both inflammation and degeneration of the plantar fascia, a fibrous connective tissue that extends from the heel to the toes and plays a critical role in foot biomechanics [2]. Several risk factors contribute to the development of PF, including prolonged standing, elevated body mass index (BMI), restricted ankle mobility, and inappropriate footwear, such as high heels [3]. Epidemiological studies estimate that plantar heel pain (PHP) affects approximately 9.6% of individuals aged 50 or older, with 7.9% experiencing disabling symptoms [4,5]. Furthermore, chronic PF often persists over time, with nearly 45.6% of patients continuing to report symptoms even a decade after onset, which significantly impacts mobility and quality of life [6].

Ankylosing spondylitis (AS), a chronic inflammatory disease primarily affecting the spine and sacroiliac joints, has been closely associated with PF [7]. Notably, AS frequently leads to enthesopathy, a condition characterized by inflammation at the insertion sites of tendons and ligaments onto bones [7]. Due to this association, the early detection of fascia involvement in AS patients is essential for timely diagnosis and intervention, potentially preventing disease progression and related disability [8]. Since AS is often progressive, identifying subtle entheseal changes in the fascia can lead to better patient outcomes.

Ultrasound (US) imaging has emerged as a valuable, non-invasive, and cost-effective modality for evaluating fascia and related enthesopathy by providing the rapid visualization of soft tissue abnormalities [5,9]. Among the available US techniques, shear wave elastography (SWE) has gained prominence as an advanced method for assessing tissue stiffness by measuring the speed of mechanical shear waves propagating through soft tissues [10]. Unlike conventional grayscale US, SWE quantifies fascia stiffness in kilopascals (Kpa), offering an objective and reproducible measurement [5,9]. This capability enables SWE to detect subclinical biomechanical changes, even in the absence of visible structural abnormalities [9,11], making it an important tool for evaluating plantar fascia integrity in both AS and non-AS populations [9,12].

The BUSES is a validated scoring system specifically designed to assess entheseal abnormalities by integrating grayscale and color Doppler US findings [13]. It evaluates key characteristics such as tendon thickness, hypoechogenicity, enthesophytes, erosion, and vascularity, offering a comprehensive assessment of both structural and inflammatory changes [14]. The BUSES has demonstrated high validity and reliability in detecting enthesopathy, particularly among AS patients [13,14]. By combining grayscale US for anatomical evaluation with Doppler findings for inflammation, the BUSES provides a detailed and holistic analysis of fasciopathy and other enthesis-related abnormalities [15].

Given these considerations, a comparative evaluation of SWE and the BUSES in PF diagnosis may clarify their respective diagnostic strengths. This investigation aims to compare the diagnostic accuracy of SWE and the BUSES in identifying PF and related functional impairment. Using controlled binary logistic regression (LR) models and evaluating key diagnostic metrics, including sensitivity and specificity, this study assesses the relative discriminative strength of both imaging techniques in AS and non-AS populations.

## 2. Materials and Methods

### 2.1. Study Design and Participants

This cross-sectional, comparative study was approved by the Institutional Review Board, and written informed consent was obtained from all participants. The study was conducted between March 2023 and February 2024 at the Rheumatology Research Center of a tertiary academic hospital. A total of 96 participants were enrolled using simple non-random sampling. The required sample size was determined based on prior studies evaluating US application for the assessment of PF [5]. Diagnosis of PF was confirmed through detailed history and physical evaluation by a rheumatologist and an orthopedic surgeon, each with over 20 years of clinical experience, applying standardized diagnostic criteria. The rheumatologist and orthopedic surgeon who confirmed clinical PF were blinded to the subsequently acquired ultrasound, BUSES, and SWE results.

Patients with AS met the modified New York criteria and reported plantar heel pain persisting for more than three months. Classification and diagnostic confirmation were performed in accordance with the Assessment of SpondyloArthritis international Society (ASAS) handbook guidelines [16]. Similarly, non-AS participants diagnosed with clinical PF also reported heel pain of greater than three months’ duration, confirmed through clinical evaluation. Exclusion criteria included alternative causes of heel pain (e.g., trauma, infection, and neoplasm), uncontrolled diabetes, prior foot surgery, congenital foot deformity, or conditions that precluded local US examination (such as skin lesions and metal implants).

Disease activity in AS population was quantified via the Bath Ankylosing Spondylitis Disease Activity Index (BASDAI), which is a self-reported questionnaire ranging from 0 to 10, with higher scores indicating higher disease activity [17]. Moreover, foot function and pain in all individuals were evaluated using the AOFAS, a validated instrument consisting of three items, i.e., pain, function, and alignment, ranging from 0 to 100, with higher scores indicating better foot function and less pain [18].

### 2.2. Group Classification

Demographic and clinical data—including age, sex, BMI, the BASDAI, and the AOFAS—were collected for all participants. Subjects were first stratified based on the presence or absence of AS, and then further categorized according to the presence or absence of clinical PF, resulting in four distinct subgroups. In the AS population, participants were categorized as either having AS without clinical PF or having both AS and clinical PF. In the non-AS population, one subgroup comprised healthy individuals without clinical PF, while the other included individuals with clinical PF but no AS. Subgroups without clinical PF served as normative references, enabling baseline comparisons of SWE and BUSES values within each population. The inclusion of healthy controls allowed for the establishment of reference standard US and SWE features of the plantar fascia in the absence of localized pathology.

### 2.3. Image Acquisition

US of the plantar fascia was performed using a high-frequency linear transducer (5–18 MHz) on a real-time system (Aixplorer Ultimate, Supersonic Imagine, Aix-en-Provence, France). Participants were scanned, with ankles relaxed, using ample coupling gel (Aquasonic 100, Parker Laboratories, Fairfield, NJ, USA); care was taken to align the beam perpendicular to the plantar fascia to minimize anisotropy [19]. Two musculoskeletal radiologists (≥5 years’ experience) independently performed all examinations, blinded to clinical PF diagnosis. The BUSES applied to quantify enthesitis-related US changes. The composite score comprises five components (tendon thickness, hypoechogenicity with fibrillar loss, enthesophytes, erosion, and power Doppler signal), producing a total score ranging from 0 to 11 [13,20].

Next, SWE was employed to objectively quantify plantar fascia stiffness as an additional imaging biomarker. SWEs were acquired by placing the transducer steadily with gentle pressure and a coupling gel for 4–5 s. A manually positioned region of interest (ROI) centered on the origin of plantar fascia yielded automatic mean stiffness values [21]. Two longitudinal images, each with three repeat acquisitions (quality factor ≥ 60), were obtained to reduce variability. The averaged value of these measurements per site was used for analysis.

### 2.4. Statistical Analysis

Statistical analysis was performed using the IBM SPSS Statistics software (version 29.0.2; IBM Corp., Armonk, NY, USA). Descriptive statistics (Table 1) were calculated for demographic and clinical variables, with continuous data summarized as mean ± standard deviation and categorical data as frequencies and percentages. Binary logistic regression was chosen because the outcome (clinical PF: yes/no) is dichotomous, the method allows adjustment for covariates (age, BMI, fascia–skin distance), and the model’s predicted probabilities can be directly used to construct ROC curves and calculate AUC, providing a unified framework for both effect estimation and diagnostic-accuracy assessment.

Binary LR analyses were conducted separately for AS and non-AS populations to evaluate the predictive values of the BUSES and SWE for diagnosing PF, while controlling for age, BMI, and fascia–skin distance. For each population, two models were constructed: one including the BUSES and the other including SWE as the sole predictor. Model performance was assessed using the omnibus chi-square test, Nagelkerke R^2^ was used to estimate the explained variance, and classification accuracy was used to evaluate predictive performance. The Hosmer–Lemeshow test was applied to assess model fit. Predictor significance was determined using Wald statistics, with adjusted odds ratios (AORs) reported alongside standard errors (SEs) and *p*-values. Models were compared based on diagnostic strength, variance explained (Nagelkerke R^2^), and classification metrics including sensitivity, specificity, and overall accuracy.

To compare diagnostic performance, receiver operating characteristic (ROC) curves were generated for the BUSES and SWE based on the existence of AS. The area under the curve (AUC) and 95% confidence interval (CI) were computed for each diagnostic modality.

Interobserver agreement between the two independent image readers was assessed using the intraclass correlation coefficient (ICC) based on a two-way random-effects model with absolute agreement. ICC values were interpreted as follows: poor (<0.50), moderate (0.50–0.75), good (0.75–0.90), and excellent (>0.90). For statistical analysis, a single value of SWE and the BUSES per subject was used, determined through a consensus meeting between readers to establish agreed-upon SWE and the BUSES values.

A *p*-value of < 0.05 was considered statistically significant. AORs with 95% CIs were reported for all regression coefficients.

## 3. Results

**Figure 1:**
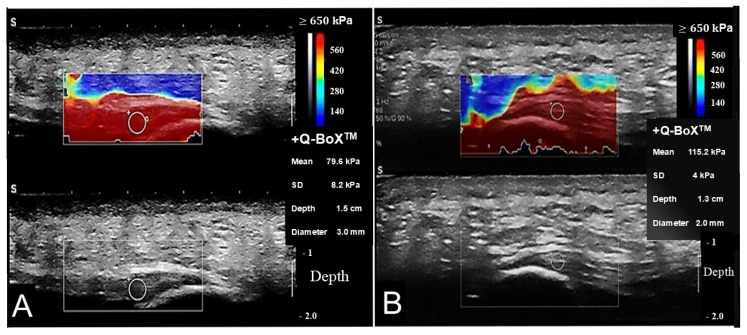
Representative longitudinal SWE and B-Mode images. The region of interest (ROI) was selected based on anatomical landmarks, as shown in the color-coded images (upper panels) and the corresponding grayscale images (lower panels). The Q-Box (white circle) was placed at the proximal portion of the plantar fascia, providing the mean, standard deviation, distance of the ROI from the skin, and diameter of the ROI. These measurements are displayed alongside each examination. The color map illustrates tissue stiffness, with red indicating the stiffest tissues and dark blue indicating the least stiff. Panel (**A**) shows images from a 56-year-old woman with ankylosing spondylitis (AS) and clinical features of plantar fasciitis, demonstrating reduced SWE values. Panel (**B**) presents images from a 39-year-old man with a known diagnosis of AS but without clinical signs of plantar fasciitis, showing normal SWE measurements.

The representative longitudinal SWE images of individuals are shown in Figure 1, demonstrating quantitative stiffness values and corresponding color maps. The ROI and the depth from the skin surface to the fascia location are also annotated. Table 1 presents the sample size and gender distribution of each group. In addition, mean and standard deviation (SD) of age, BMI, the AOFAS, and the BASDAI are provided.

### 3.1. Binary Logistic Regression Analysis

In the AS population, the first model (Table 2, row 1) with SWE remained significant while controlling for age, BMI, and fascia–skin distance. The LR, χ^2^(4) = 16.297, *p* = 0.003, explaining 44.6% of outcome variance (Nagelkerke R^2^ = 0.44). The SWE model achieved 80% classification accuracy, with good fit confirmed by the Hosmer–Lemeshow test (χ^2^ = 9.64, *p* = 0.29). SWE was a strong and significant predictor (B = 1.611, SE = 0.580, *p* = 0.005; Exp(B) = 5.010). In the next model (Table 2, row 2), the BUSES was entered as the sole predictor along with confounders. The LR model demonstrated statistically significance, χ^2^(4) = 14.74, *p* = 0.005, accounting for 41.1% of the variance in the outcome (Nagelkerke R^2^ = 0.41). The model demonstrated an overall classification accuracy of 75%, with balanced accuracy across outcome categories. The Hosmer–Lemeshow test supported an adequate model fit (χ^2^ = 6.31, *p* = 0.61). The BUSES was a strong and statistically significant predictor of PF, with B = 1.51 (SE = 0.573), Wald = 6.97, *p* = 0.008, and an AOR of 4.53.

In the non-AS population, LR model using SWE (Table 2, row 3) as the primary predictor along with confounders as covariates yielded a statistically significant model (χ^2^(4) = 42.634, *p* < 0.001), explaining 71.2% of the variance (Nagelkerke R^2^ = 0.712). The model demonstrated high classification accuracy (83.9%), with 80.8% sensitivity and 86.7% specificity. Therefore, SWE is a significant predictor of PF (B = 1.459, SE = 0.511, Wald = 8.162, *p* = 0.004), with an AOR of 4.30. In the second model (Table 2, row 4), the BUSES produced a statistically significant LR (χ^2^(4) = 39.238, *p* < 0.001), accounting for 67.3% of the variance in outcome (Nagelkerke R^2^ = 0.673). The model exhibited high classification accuracy (89.3%), with 88.5% sensitivity and 90.0% specificity. The BUSES was identified as a significant predictor of PF (B = 1.344, SE = 0.596, Wald = 5.084, *p* = 0.024), with an AOR of 3.833.

### 3.2. ROC Analysis

**Figure 2:**
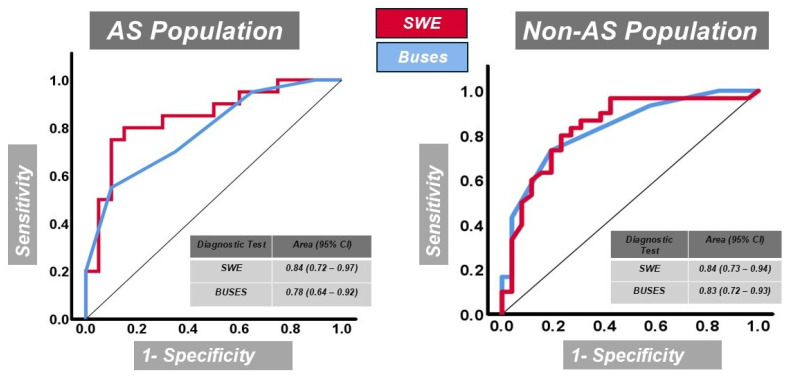
ROC curves comparing the diagnostic performance of SWE and BUSES for detecting plantar fasciitis in the AS population (left panel) and the non-AS population (right panel). The black diagonal reference line represents random chance or no diagnostic discrimination (AUC = 0.50). Curves positioned above this diagonal indicate better diagnostic accuracy. The AUC with the 95% confidence interval (CI) for each modality is provided within each panel.

In the AS population, ROC analysis (Figure 2) demonstrated significant discrimination by both SWE and the BUSES (*p* < 0.001). SWE achieved superior diagnostic performance with an AUC of 0.845 (SE = 0.064; 95% CI: 0.720–0.970), compared to the BUSES with an AUC of 0.785 (SE = 0.071; 95% CI: 0.645–0.925). The analysis was based on 40 subjects, evenly distributed between positive and negative outcomes. Furthermore, in the non-AS population, ROC analysis (Figure 2) revealed that both SWE and the BUSES significantly distinguished between positive and negative outcomes (*p* < 0.001). SWE exhibited slightly superior diagnostic accuracy, with an AUC of 0.837 (SE = 0.056; 95% CI: 0.726–0.947), while the BUSES produced an AUC of 0.831 (SE = 0.054; 95% CI: 0.724–0.938). The analysis included 56 participants, comprising 30 positive and 26 negative cases. These findings suggest that SWE provides slightly greater diagnostic accuracy for PF in the AS group, possibly due to its sensitivity to early biomechanical changes.

Interobserver agreement for SWE measurements was good, with an ICC of 0.81 (95% CI: 0.74–0.88), while the BUSES showed slightly better reliability, with an ICC of 0.88 (95% CI: 0.83–0.93).

## 4. Discussion

This study investigated the diagnostic accuracy of SWE and the BUSES in identifying clinical PF across AS and non-AS populations while controlling confounding factors. SWE demonstrated slightly superior AUC values and adjusted odds ratios, particularly in the AS population. Additionally, the BUSES, as a tool for assessing anatomical and vascular features, also performed effectively, especially in the non-AS group. Integrating both SWE and the BUSES within a multimodal imaging approach allows for a comprehensive evaluation, with SWE facilitating early detection and the BUSES contributing to structural confirmation. This combined strategy provides a robust diagnostic framework adaptable to patient-specific variables such as age, body composition, and anatomical variation.

Our investigations support the idea that SWE can be valuable in identifying plantar fasciopathy apart from anatomical degradation. This highlights the value of soft-tissue assessment in identifying pathology before anatomical changes appear. Interestingly, combining soft tissue stiffness assessment by SWE and anatomical assessment by the BUSES in a multimodal US approach provides a comprehensive evaluation of fasciopathy. Consistent with our findings, Baraliakos et al. also reported benefits in using US for detecting enthesopathy and structural changes in spondylarthritis [22]. Consistent with our results, Carotti et al. highlighted the added value of advanced ultrasound techniques in detecting subtle inflammatory changes that are often missed on conventional grayscale imaging [23]. Moreover, the increased detection of PF compared to controls by SWE, which has been found in recent studies, reinforced our hypothesis of the possible role of SWE in identifying subclinical PF [21]. By detecting early compositional changes, SWE may facilitate timely intervention, potentially prevent disease progression, and enhance patient care.

In our study, the BUSES found few imaging markers among participants without clinical PF such as enthesophyte, a loss of normal fibrillary pattern, and less likely increased thickness (above 2 mm). Enthesophyte may be seen among individuals without the clinical evidence of PF. Similarly, variations in the plantar fascia US patterns have been illustrated in the literature. The normal range of plantar fascia thickness could also be a misleading US finding for sonographers who are unaware of clinical findings [24]. These findings may lead clinicians to be cautious on making decisions solely by the crude BUSES. However, it is important to acknowledge that the BUSES has been extensively validated in previous research [25,26]. For instance, Florescu et al. confirmed its effectiveness in assessing enthesopathy in spondylarthritis [13], while Milutinovic et al. demonstrated its correlation with clinical symptoms of PF [14]. Furthermore, prior studies have reported a positive correlation between BUSES and PF severity, confirming its specificity and utility in identifying AS-related enthesopathy [25]. These observations further support integrating SWE with conventional scoring systems.

In our regression model, we consider age as a confounder to control age-related changes in the anatomical features and elasticity. Although the impact of age on tendon elasticity was not the primary focus of this study, previous research by Delabastita et al. and Kwan et al. suggests that aging affects tendon elasticity and plantar fascia biomechanics, making older individuals more prone to stiffness and plantar fasciitis [27,28]. Additionally, we considered subcutaneous fat thickness (plantar fascia–skin distance) and BMI as confounding factors in our study design to control for the possible elasticity variations secondary to these factors. The increased mechanical stress and the pro-inflammatory effects of obesity are known contributing factors [29]. Franceschi et al. explored the impact of obesity on tendon structures, while Wearing et al. examined how excess weight contributes to plantar fascia abnormalities and related musculoskeletal disorders [30,31]. While SWE and the BUSES remain reliable diagnostic tools across different patient demographics, clinicians should carefully consider the influence of age and BMI when interpreting results.

The BASDAI scores were significantly higher in AS patients with PF than AS subjects with no PF, reflecting greater disease activity and systemic inflammation. Aligning with it, the AOFAS was lower among AS patients with clinical PF, indicating poor foot function and increased pain. These observations agree with the results of Redeker et al., who also found higher disease activity and functional impairment in AS patients with active enthesitis, such as PF [32]. Similarly, Wärnberg et al. reported that disease activity scores were linked to reduced functional capacity in AS patients, reinforcing the role of systemic inflammation in limiting physical performance [33].

Despite these findings, our study has several limitations. First, the relatively small sample size may limit the generalizability of the results. Second, the study population was drawn from a specific geographic location, potentially affecting its applicability to broader populations. Third, we did not perform baseline and follow-up SWE assessments, preventing us from analyzing longitudinal changes in plantar fascia stiffness over time. This limitation precludes the assessment of SWE’s utility in monitoring disease progression or response to treatment. Consequently, our cross-sectional study design restricted us from developing a model to show how SWE measurements evolve with disease progression.

## 5. Conclusions

Both SWE and the BUSES proved effective in diagnosing PF in AS and non-AS populations. While the BUSES performed well, its interpretation may be confounded by normal variants such as enthesophytes and altered fascial patterns. Therefore, SWE may offer value in detecting soft tissue changes beyond equivocal US anatomical observations. Given its ability to detect subclinical changes, SWE could be incorporated into early diagnostic protocols for enthesopathy, especially in inflammatory rheumatic disorders.

## Figures and Tables

**Table 1:** Demographic and clinical indices of subjects based on underlying disease and clinical presence of PF.

Subjects	Clinical PF	Sample Size	Sex (male%)	Age	BMI (kg/m^2^)	AOFAS	BASDAI
AS Population	No	20	46%	44 ± 9	26 ± 4	88 ± 10	3.25 ± 1
	Yes	20	54%	39 ± 11	26 ± 3	71 ± 14	5.39 ± 2
Non-AS Population	No	26	47%	41 ± 10	25 ± 2	92 ± 4	N/A
	Yes	30	53%	47 ± 11	28 ± 3	74 ± 11	N/A

PF: plantar fasciitis; BMI: body mass index; AOFAS: American Orthopedic Foot and Ankle Society Score; BASDAI: Bath Ankylosing Spondylitis Disease Activity Index.

**Table 2:** Binary Logistic Regression Analysis: models are designed to predict the possibility of clinical PF based on imaging technique and population.

Subject	Model	Adjusted Odds Ratio	95% CI	*p*-Value
AS Population	SWE	5.00	1.61–15.61	0.005
	BUSES	4.53	1.48–13.94	0.008
Non-AS Population	SWE	4.30	1.58–11.70	0.004
	BUSES	3.83	1.19–12.32	0.024

SWE: shear wave elastography; BUSES: Belgrade ultrasound enthesitis score; CI: confidence interval.

## Data Availability

The data of study is available from corresponding authors upon request.

## Abbreviations

The following abbreviations are used in this manuscript:

PF	Plantar fasciitis
AS	Ankylosing spondylitis
US	Ultrasound
kPa	Kilopascals
SWE	Shear wave elastography
BUSES	Belgrade ultrasound enthesitis score
LR	Logistic regression
ASAS	Assessment of spondyloarthritis international society
BASDAI	Bath ankylosing spondylitis disease activity index
AOFAS	American orthopedic foot and ankle society score
AOR	Adjusted odds ratio
ROI	Region of interest
AUC	Area under the curve
CI	Confidence interval
ICC	Intraclass correlation coefficient
SE	Standard error
